# Endoplasmic Reticulum Stress and Diabetic Cardiomyopathy

**DOI:** 10.1155/2012/827971

**Published:** 2011-11-24

**Authors:** Jiancheng Xu, Qi Zhou, Wei Xu, Lu Cai

**Affiliations:** ^1^Department of Clinical Laboratory at the First Bethune Hospital, Jilin University, Changchun 130021, China; ^2^Department of Pediatrics at the First Bethune Hospital, Jilin University, Changchun 130021, China; ^3^Department of Pediatrics, University of Louisville, Louisville 40202, KY, USA

## Abstract

The endoplasmic reticulum (ER) is an organelle entrusted with lipid synthesis, calcium homeostasis, protein folding, and maturation. Perturbation of ER-associated functions results in an evolutionarily conserved cell stress response, the unfolded protein response (UPR) that is also called ER stress. ER stress is aimed initially at compensating for damage but can eventually trigger cell death if ER stress is excessive or prolonged. Now the ER stress has been associated with numerous diseases. For instance, our recent studies have demonstrated the important role of ER stress in diabetes-induced cardiac cell death. It is known that apoptosis has been considered to play a critical role in diabetic cardiomyopathy. Therefore, this paper will summarize the information from the literature and our own studies to focus on the pathological role of ER stress in the development of diabetic cardiomyopathy. Improved understanding of the molecular mechanisms underlying UPR activation and ER-initiated apoptosis in diabetic cardiomyopathy will provide us with new targets for drug discovery and therapeutic intervention.

## 1. Introduction

The endoplasmic reticulum (ER) is a central organelle entrusted with lipid synthesis, calcium homeostasis, protein folding, and maturation [1]. Most secreted and integral membrane proteins of eukaryotic cells are translocated into the lumen of ER. The ER lumen provides a specialized environment for posttranslational modification and folding of secreted, transmembrane, and resident proteins of the various compartments of the endomembrane system [2]. Various factors that interfere with ER function lead to accumulation of unfolded proteins, including oxidative stress, ischemia, disturbance of calcium homeostasis, and overexpression of normal and/or incorrectly folded proteins. This process is called ER stress and further activates the unfolding protein response (UPR). There are two aims of the UPR: the UPR initially tries to restore normal function of the cell by halting protein translation and activating the signaling pathways that lead to increasing the production of molecular chaperones involved in protein folding; if these objectives are not achieved within a certain time lapse or the disruption is prolonged, the UPR tries to turn on apoptotic pathway [3–6]. Therefore, the UPR can be considered as a safeguard for protein synthesis, posttranslational modifications, folding and secretion, calcium storage and signaling, and lipid biosynthesis [7–11]. Under normal conditions the ER maintains high concentrations of resident calcium-dependent chaperone proteins, such as glucose-regulated protein-78 (GRP78, also known as BiP) and glucose-regulated protein-94 (GRP94) [12], along with a high level of calcium and an oxidized environment. Only properly folded proteins are allowed to reach their final destination, whereas unfolded and misfolded proteins are exported or dislocated from the ER and degraded by cytoplasmic proteasomes. The stresses that can cause the UPR include a variety of conditions such as elevated secretory protein synthesis, overexpression and/or accumulation of mutant proteins, aberrant Ca^2+^ regulation, hypoxia, glucose deprivation, altered glycosylation, ischemia, ER calcium depletion, viral infections, shifting of redox status to a more reduced state, exposure to natural and experimental toxins that perturb ER function, and overloading of cholesterol [3, 10, 13–17].

In human and animal models of diabetes, a heart muscle-specific disease in the absence of other vascular pathology has been described, termed diabetic cardiomyopathy [18, 19]. The pathogenesis of diabetic cardiomyopathy is a chronic and complex process that is attributed to abnormal cellular metabolism and defects in organelles such as myofibrils, mitochondria, and sarcolemma [20–24]. Probable candidates to explain this heart disease include autonomic abnormalities, metabolic disorders, abnormal enzyme function, and interstitial fibrosis [25–27]. For instance, apoptosis, as a regulated, energy-dependent, cell suicide mechanism has also been reported to play a critical role in the development of diabetic cardiomyopathy [28–31].

Recently the involvement of ER stress in the development of diabetic cardiomyopathy was also reported [32, 33]. However, the mechanisms of diabetic cardiomyopathy are not fully known, and appropriate approaches to minimize these risks are still being explored. This paper focuses on ER stress and its involvement in the development of diabetic cardiomyopathy in order to provide certain new insights into understanding of its mechanisms.

## 2. Adaptation to ER Stress: Mechanisms to Restore Homeostasis

The ability to adapt to physiological levels of ER stress is important to cells. The initial intent of the UPR is adaptation and restoration of the normal ER function. When unfolded proteins accumulate in the ER, resident chaperones become occupied, releasing transmembrane ER proteins involved in inducing the UPR. These proteins straddle ER membranes, with their N-terminus in the lumen of the ER and their C-terminus in the cytosol, providing a bridge that connects these 2 compartments. The N-termini of these transmembrane ER proteins are usually associated with or bound to intralumenal GRP78 in the absence of ER stress, preventing their aggregation. In some physiological or pathological conditions, the large excess of unfolded proteins in the ER lumen necessitates GRP78 dissociation and launches the UPR. The UPR initially activates intracellular signaling pathways mediated by three ER-resident proteins in mammalian cells: the inositol-requiring kinase-1 (IRE-1) [34, 35], the activating transcription factor 6 (ATF6), and the protein kinase R-like ER kinase (PERK) [36, 37].

### 2.1. PERK

**Figure 1:**
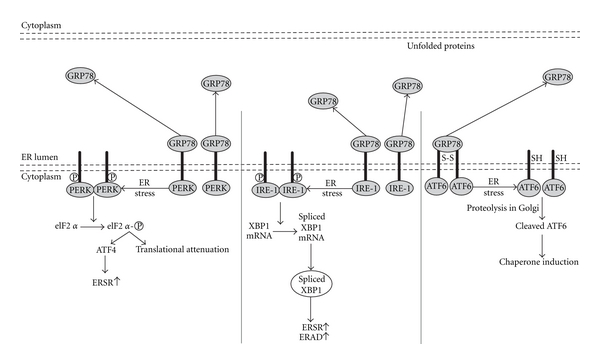
ER stress signaling pathways.

The UPR induces an early and transient attenuation of protein biosynthesis which is mediated by PERK, an ER-resident protein whose effector the eukaryotic initiation factor 2*α* kinase (eIF2*α*) domain lies on the cytoplasmic side of the ER membrane and whose stress-sensing domain lies on the opposite side of the membrane in the ER lumen [38], as illustrated in Figure 1. The luminal domain of monomeric PERK binds with the ER chaperone GRP78 in an inactive complex under unstressed conditions. However, as client proteins accumulate in the ER lumen, in efforts to assist folding, GRP78 relocates from PERK to misfolded ER proteins. GRP78 relocalization allows PERK to dimerize, which facilitates transautophosphorylation [39, 40]. PERK is then activated, and it phosphorylates *α*-subunit of eIF2*α* on serine-51. This phosphorylation event decreases cap- or eIF2*α*-dependent translation, which shuts off global mRNA translation and reduces the protein load on the ER [37, 39]. Global translational inhibition acutely reduces the protein-folding load on the ER and allows the cell to focus resources on resolving the ER stress, thus facilitating survival. However, certain mRNAs encoded by ER stress response (ERSR) genes gains structural features and a selective advantage for translation that allow them to escape PERK-mediated translational inhibition [41]. For example, eIF2*α* phosphorylation induces expression of GRP78 and ATF4 under stress [42, 43]. During the prosurvival phase of ER stress, ATF4 induces numerous genes involved in resolution of the ER stress, such as genes that encode amino acid transporters and ER-resident chaperones [43]. However, after prolonged ER stress, continued ATF4 expression mediates the upregulation of genes that contribute to programmed cell death (Figure 1).

### 2.2. IRE-1

Chaperone induction and ER-associated degradation (ERAD), in response to ER stress, are regulated by the IRE-1 pathway. IRE-1 contains both a Ser/Thr kinase domain and an endoribonuclease domain, which thus functions as a kinase and an endoribonuclease. Like PERK, under normal conditions, the luminal domain of IRE-1 monomers associates with GRP78. Also in comparison with PERK, during the prosurvival phase of the ERSR, GRP78 relocates to misfolded proteins, which allows IRE-1 to dimerize, thus facilitating transautophosphorylation [44]. However, in contrast to PERK, transautophosphorylation of IRE-1 activates a novel endoribonuclease activity [45]. In mammalian cells, the substrate for the IRE-1 endoribonuclease is the X-box-binding protein-1 (XBP1) mRNA. After activation, the IRE-1 endoribonuclease splices the XBP1 mRNA with an altered reading frame. This XBP1 splice variant binds to the promoters containing ER stress response elements (ERSEs) [46]. These findings suggest that ER stress, acting through the IRE-1- and XBP1-dependent signaling pathway, upregulates the secretory apparatus in cells. Defective signaling in this pathway would affect professional secretory cells (Figure 1).

### 2.3. ATF6

ATF6 is an ER transmembrane protein [47, 48]. Two similar transcription factors, ATF6*α* and ATF6*β*, exist in mammals. In comparison to PERK and IRE-1, in normal cells, the luminal domain of ATF6 is associated with GRP78 in an inactive form. It should be mentioned that even though ER stress releases GRP78 from ATF6, in contrast to PERK and IRE-1, this is not thought to be attributable to competitive binding of GRP78 to other proteins [49]. Moreover, ATF6 exists in the ER as a dimer linked by intermolecular disulfide bonds in the luminal domain. GRP78 dissociation and disulfide bond cleavage on ER stress facilitate the translocation of ATF6 to the Golgi [50], where it undergoes cleavage by site-1 and site-2 proteases. This yields N-ATF6 that translocates to the nucleus and induces target ER genes [36, 48, 51–58]. Therefore, activation of ATF6, IRE-1, and the downstream XBP1 (IRE-1-XBP1) increases the expression of ER-resident chaperones. The genes induced by ATF6 during the prosurvival phase of ER stress foster resolution of the stress and, thus, survival, whereas those genes induced on the proapoptotic phase of ER stress contribute to programmed cell death [59] (Figure 1).

### 2.4. Apoptosis Induced by ER Stress

**Figure 2:**
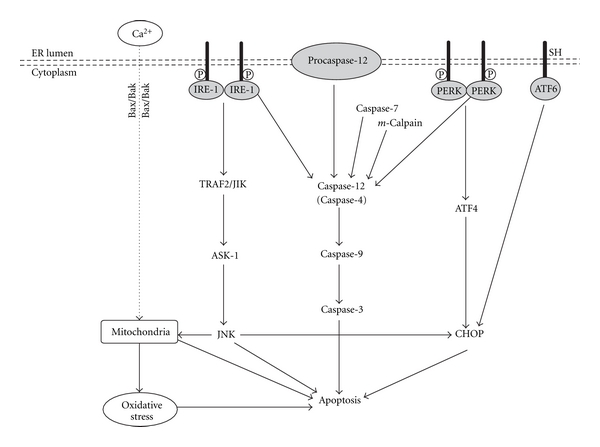
Apoptosis mechanisms induced by ER stress.

The UPR deals with adverse effects of ER stress in a timely and efficient manner at the early stage and thus enhances cell survival. However, when protein misfolding is persistent or excessive, prolonged ER stress has severe consequences, including apoptosis. When severe and prolonged ER stress extensively impairs the ER functions, apoptosis is necessary not only for removing the cells that threaten the integrity of the organism but also for proper development and differentiation [1]. Although the induction of apoptosis is the least well understood among the responses to ER stress, the apoptotic mechanisms induced by ER stress remain able to be broadly divided into a few categories, as summarized in Figure 2.

The first apoptotic pathway involves activation of the c-Jun N-terminal kinase (JNK) pathway. During ER stress, activated IRE-1 recruits tumor necrosis factor receptor-associated factor 2 (TRAF2) and apoptosis signal-regulating kinase 1 (ASK-1) to form IRE-1-TRAF2-ASK1 complex which then lead to activation of JNK and downstream mitochondria/Apaf-1-dependent caspase activation [60, 61]. Also, the c-Jun-N-terminal inhibitory kinase (JIK) interacts with activated IRE-1 and promotes phosphorylation and association of TRAF2 with IRE-1 (Figure 2).

The second apoptotic pathway depends on the activation of ER-localized cysteine protease, caspase-12 in rodents [62]. Several processes have been suggested as contributing factors to caspase-12 activation in ER-stressed cells. *m*-Calpain, a cysteine protease activated by disturbed calcium homeostasis in ER-stressed cells, may directly cleave and activate caspase-12 [63]. Caspase-7, which is translocated from cytosol to the ER surface in stressed cells, can cleave and activate caspase-12 [64]. ER stress-activated IRE-1 and PERK may lead to clustering caspase-12 at the ER membranes by recruitment of TRAF2 proteins [60]. Upon activation, in rodents but not in humans, caspase-12 translocates from the ER to the cytosol, where it directly cleaves procaspase-9, which, in turn, activates the downstream effector caspase, caspase-3 without the need for mitochondrial amplification [62, 65]. Therefore, caspase-12-mediated apoptosis was a specific apoptosis pathway of ER, which is independent on mitochondria or death receptor activation. Caspase-4, one of the closest paralogs of rodent caspase-12, has been suggested to fulfill this role normally ascribed to rodent caspase-12 in the context of ER stress in human [66] (Figure 2). In a recent study, the enhanced expression of the cleaved caspase-12 as an indicator of ER stress-associated apoptosis was also observed in the diabetic heart [32].

The third apoptotic pathway activated by ER stress is mediated by transcriptional activation of CHOP/GADD153, a member of the C/EBP family of b-ZIP transcription factor that potentiates apoptosis, possibly through repressing expression of antiapoptotic Bcl2 and Bcl-X_L_ and induction of ER oxidase 1*α* which generates reactive oxygen species (ROS) and depletes glutathione (GSH) [67]. While CHOP is barely detected under physiological conditions, it is strongly induced in response to ER stress [68]. Although both the IRE-1 and ATF6 pathways can upregulate CHOP, the PERK pathway predominates through selective upregulation of translation of ATF4, which subsequently induces transcription of CHOP and other genes involved in amino acid metabolism and transport, and oxidation-reduction reactions [69, 70]. The downstream targets of CHOP leading to apoptosis are still unclear (Figure 2).

In addition, prolonged ER stress is associated with release of ER Ca^2+^ stores which can perturb mitochondria, triggering oxidative stress. Ca^2+^-induced oxidative stress can induce cell death. Increased cytosol Ca^2+^ also activates calpains, a family of Ca^2+^-dependent cysteine proteases which proteolytically cleave caspase-12 (activated), Bcl2, and Bcl-X_L_ (inhibited). Apoptosis is rapidly initiated after ER-Ca^2+^ depletion in photodynamic therapy and strictly requires Bax/Bak at the mitochondria [71] (Figure 2).

## 3. ER Stress in the Heart

### 3.1. Requirement of the ER Stress for the Heart

Several studies have indicated that the ER stress is required for the proper cardiac differentiation and development. It is reported that many genes encoding ER-resident proteins are activated during the early stages of cardiogenesis. For instance, GRP78 can be activated to promote early heart organogenesis through cooperation between the cell type-specific transcription factors and ERSE-binding factors [72]. GRP78 deficiency is lethal at a very early stage of embryogenesis [73]. GRP94 knockout cells fail to develop mesoderm, resulting in a prevention of cardiac development from beginning [74]. Targeted disruption of the XBP1 gene in mice is embryonic lethal because of cardiac development defects [75]. All these studies clearly confirm the requirement of ERSR for the proper cardiac development.

ERSR also involves in the cardiac protection against certain challenges. For instance, GRP78 antisense oligodeoxynucleotide partially abrogated the protective effect of endothelin-1 pretreatment on hypoxic cardiomyocyte injury [76]. Overexpression or pharmacological induction of GRP78 can attenuate cardiomyocyte death induced by proteasome inhibition [77]. Similarly, overexpression of GRP94 also reduces cardiac cell death caused by calcium overload or ischemia [78]. In cardiac ischemia/reperfusion injuries model, ATF6 transgenic hearts exhibited a better recovery of ventricular pressure and lower incidence of cardiac cell death [79]. A recent study showed that induction of autophagy by ER stress before ischemia (similar to ischemic preconditioning) could reduce ischemia/reperfusion-induced lethal injury [80]. Therefore, the ER stress is not only required for the cardiac development, but also provide certain protective mechanisms for the heart against damage caused by various stresses.

### 3.2. The Deleterious Effect of the ER Stress on the Heart

However, ER stress is demonstrated to be pathologically involved in cardiac diseases and damages under numerous conditions, including myocardial infarction, ischemia/reperfusion, and pressure overload. For example, the ERSR is activated in the hearts of transgenic mice that overexpress monocyte chemoattractant protein-1 (MCP-1) and develop heart failure [81]. They found that the heart failure is mainly due to the activated proapoptotic phase of ERSR that led to massive loss of cardiomyocytes [81]. A further support of a role for ER stress in heart failure is the finding that transgenic overexpression of a mutant KDEL receptor, a retrieval receptor for ER chaperones in the early secretory pathway, induced the ERSR in the hearts, in parallel with a consequence of dilated cardiomyopathy [82].

Hypoxia is an insult that activates cardiac ER stress. During ischemia that could result from the reduced availability of molecular oxygen, significant changes in cardiac energy metabolism happen [83]. Under severe hypoxia, anaerobic metabolism has to occur and results in a massive increase in ROS, leading to cardiac oxidative damage, cell death, and, ultimately, cardiac dysfunction [84]. In addition, reperfusion after ischemia generates increased oxidative stress as the heart converts back to aerobic respiration, thereby generating lethal levels of ROS. Ischemia/reperfusion injury in the heart results in numerous cellular and molecular events that lead to loss of cardiac damage and dysfunction, such as disrupting ER oxidative state or Ca^2+^ homeostasis, triggering cellular damage, and eventually causing cardiac apoptotic death [84, 85].

Apoptotic cell death as an early cellular event in response to diabetes has been reported to play a critical role in the development of diabetic cardiomyopathy [28–30]. Because myocytes rarely proliferate in adult cardiac muscles, the loss of cardiomyocytes would eventually lead to compromised cardiac function. Loss of endothelial cells will lead the vascular system to dysfunction and aggravate the ischemia of the heart. Apoptosis of cardiomyocytes and endothelial cells has been observed in the heart of patient with diabetes and in streptozotocin- (STZ-) induced diabetic mice and rats [27, 29, 86]. Pieces of evidence have demonstrated that apoptosis induced by ER stress was involved in pathogenesis of diabetic and nondiabetic heart failure [87–89]. UPR and ER-initiated apoptosis coexist in failing hearts and the CHOP-dependent cell death pathway may be involved in the transition from cardiac hypertrophy to heart failure in mice [90]. Recent studies have also demonstrated the UPR and activation of ER-initiated apoptotic signaling in models of autoimmune cardiomyopathy [91] and alcoholic cardiomyopathy [92]. Overexpression of the ERSR gene product, p53-upregulated modulator of apoptosis (PUMA), contributes to ER stress-mediated apoptosis in cultured cardiomyocytes [93] and targeted deletion of PUMA in mouse hearts attenuates cardiomyocyte death during Langendorff ex vivo ischemia/reperfusion [94]. All these studies demonstrated that apoptotic death of cardiac cells plays a critical role of the development of diabetic cardiomyopathy, whether the cardiac cell death at diabetic early stage is mediated by diabetic induction of the ER stress still needs to be further studied.

## 4. Contribution of the ER Stress to Diabetic Cardiomyopathy

As early as 1985, whether the ER stress may play an important role in the development of diabetic cardiomyopathy has been questioned since in the diabetic hearts the ERs become swollen under ultrastructural examination [95, 96], suggesting the disorder of the ER under diabetic condition. In 2007, Li et al. provided the experimental evidence for the involvement of the ER stress in the cardiac apoptosis in a STZ-induced type 1 diabetic rat model [32]. They examined the heart function with echocardiography, morphological changes with hematoxylin-eosin staining, and cardiac cell death with TUNEL staining. Immunohistochemistry, Western blot, and real-time PCR methods were used to examine two ER stress hallmarks, GRP78 and caspase-12. They found that GRP78 and caspase-12 were upregulated at both protein and mRNA levels in the diabetic hearts compared to normal hearts. Since apoptosis plays critical role in diabetic cardiomyopathy [28, 30], those results suggested that the ER stress was induced in the diabetic hearts, and the ER stress-associated apoptosis took part in the pathogenesis of diabetic cardiomyopathy. We have also demonstrated the cardiac up-regulation of several ERSR proteins, including PERK- and ATF6-mediated pathways in diabetic hearts of mice model induced by multiple low doses of STZ (MLD-STZ). However, these ERSR were not observed in the diabetic mice with cardiac-specific overexpression of metallothionein (MT) gene (MT-TG) [86]. Since we have demonstrated that diabetes-induced cardiac cell death was prevented by MT at the diabetic early stage, resulting in a significant prevention of cardiac remodeling and dysfunction at the 5 or 6 months, that is, a significant prevention of diabetic cardiomyopathy. Based on that study we further assumed that MT prevented diabetic cardiomyopathy via suppression of diabetic induction of ER stress and associated cell death. To define the direct protection by MT from ER stress-mediated apoptotic cell death, both MT-TG mice and the age-matched wild-type (WT) mice were administrated with chemical ER stress inducer, tunicamycin. We found that cardiac ERSR such as *p*-eIF2, cleaved ATF6 and GRP78 and cardiac cell death all were significantly upregulated in tunicamycin-treated WT mice, but not in tunicamycin-treated MT-TG mice [86].

We have approved that Ang II plays a critical role in the development of diabetic cardiomyopathy [97]; therefore, we have explored and demonstrated that the exposure of embryonic rat heart-derived cells (H9c2) to induced a significant ERSR, effect abolished by MT in Ang II-treated MT-TG mice [86]. The direct role of Ang II in the induction of cardiac ERSR was also approved by a recent study that olmesartan treatment downregulated the cardiac expressions of GRP78 and caspase-12, along with oxidative and nitrosative damage [98]. To support this study, Wu et al. reported that valsartan, another selective AT1 receptor antagonist, could relieve the ER stress along with cardiomyocyte apoptosis, resulting in a significant prevention of cardiac remodeling [99].

It is well appreciated now that in addition to hyperglycemia and Ang II, diabetic heart experiences many other conditions that can invoke ER stress, such as increased oxidative stress, hypoxia, homocysteine, lipid deposition, and increased synthesis of secretory proteins [100–102]. A recent study reported the role of homocystein- (Hcy-) induced ER stress in diabetic cardiomyopathy [103]. Since significant increase in Hcy has been indicated in the development of diabetic cardiomyopathy, whether its pathogenic effect on diabetic heart is also related to ER stress has been addressed using a rat model of Hcy. These rats were given overloaded methionine to induce high-plasma Hcy. In the heart of these rats, there were significant increases in GRP78, CHOP, and caspase-12, suggesting the existence of cardiac ER stress induced by Hcy that also plays a critical role in the development of diabetic cardiomyopathy [103].

## 5. Potential Mechanisms by Which the ER Stress Causes the Development of Diabetic Cardiomyopathy

The molecular mechanisms by which hyperglycemia causes cell death are probably related to ROS production. ROS is mainly produced by mitochondria and NADPH oxidase in cardiomyocytes [29, 104]. We have used MT transgenic model to indicate the importance of oxidative stress in the induction of ER stress in the diabetic heart. Since MT is a potent, nonspecific antioxidant that can scavenge multiple ROS and/or RNS [104–106], we have examined whether diabetes induces cardiac ER stress and, if so, whether MT can prevent diabetic induction of the ER stress, resulting in a prevention of cardiomyopathy as we observed in previous studies [30, 107, 108]. Therefore we used STZ to induce diabetes in both MT-TG and WT mice. Two weeks, and 2 and 5 months after diabetes onset, cardiac ER stress was detected by expression of ER chaperones, and apoptosis was detected by CHOP, cleaved caspase-3 and caspase-12. Cardiac apoptosis in the WT diabetic mice, but not in MT-TG diabetic mice, was significantly increased 2 weeks after diabetes onset [86]. In parallel with apoptotic effect, significant up-regulation of the ER chaperones, including GRP78, GRP94, cleaved ATF6 and phosphorylated eIF2*α*, in the hearts of WT, but not MT-TG diabetic mice. Pretreatment with antioxidants completely prevented Ang II-induced ER stress and apoptosis in the cultured cardiac cells. Therefore, our results suggested that ER stress exists in the diabetic heart, which may cause the cardiac cell death. MT prevents both diabetes-induced cardiac ER stress and associated cell death most likely via its antioxidant action, which may be responsible for MT prevention of diabetic cardiomyopathy [86].

In consistence with our finding, recently, Younce et al. [109] also reported that high glucose induces cardiomyocyte death via production of MCP-1 and induction of MCP-1-induced protein (MCPIP) that results in ROS production, leading to ER stress to cause autophagy and eventual cell death. Selective inhibition of Rac1 or NADPH oxidase prevents ER stress by blocking ROS production in high-glucose-stimulated cardiomyocytes [110].

Diabetes may impair ERSR so that certain functions that can be observed in nondiabetic condition may not be observed in diabetic condition. For instance, under nondiabetic condition, pretreatment with erythropoietin (EPO) can prevent ischemia/reperfusion-induced cardiac damage, but the ER stress in diabetic hearts abolished EPO-induced cardiac protection by impairment of phospho-glycogen synthase kinase-3*β*- (GSK-3*β*-) mediated suppression of mitochondrial permeability transition [111]. In this study, the authors used type 2 diabetic (OLETF) rats and its control (LETO) to be treated with tauroursodeoxycholic acid (TUDCA) to induce ER stress. Infarction was induced by 20 min coronary occlusion and 2 h reperfusion. Levels of ER chaperones (GRP78 and GRP94) in the heart and level of non-phoshopho-GSK-3*β* in the mitochondria were significantly higher in OLETF than in LETO rats. TUDCA normalized levels of GRP78 and GRP94 and mitochondrial GSK-3*β* in OLETF rats. Administration of EPO induced phosphorylation of Akt and GSK-3*β* and reduced infarct size in LETO hearts. However, neither phosphorylation of Akt and GSK-3*β* nor infarct size limitation was induced by EPO in OLETF rats. The threshold for mitochondrial permeability transition pore (mPTP) opening was significantly lower in mitochondria from EPO-treated OLETF rats than in those from EPO-treated LETO rats. Therefore, disruption of protective signals leading to GSK-3*β* phosphorylation is due to the increased ER stress that inhibited EPO-induced suppression of mPTP opening and cardioprotection in diabetic hearts [111].

There was a recent study that has addressed how glucose induces ER stress in cardiac cells [109]. They found that glucose-induced cardiomyocyte death is mediated via MCP-1 production and MCPIP, which causes sequential events—ROS production, ER stress, autophagy, and cell death [109]. This is an elegant study, in which H9c2 cardiomyoblasts treated with 28 mmol/L glucose were evaluated for MCP-1 production and induction of MCPIP. They disrupted MCP-1 to interact with its receptor, CCR2, and knocked down MCPIP with siRNA to determine if MCP-1 and MCPIP mediate glucose-induced cell death glucose treatment of H9c2 cardiomyoblasts and isolated cardiomyocytes caused MCP-1 production, MCPIP induction, ROS production, ER stress, autophagy, and cell death. Treatment with CCR2 antagonists and knockdown of MCPIP attenuated glucose-induced ROS production, ER stress, autophagy, and cell death. Inhibition of ROS with antioxidants attenuated ER stress, autophagy, and cell death. Specific inhibitors of ER stress and knockdown of IRE-1 attenuated glucose-induced autophagy and cell death. Inhibitors of autophagy and knockdown of beclin-1 attenuated glucose-induced death. Therefore, they have demonstrated a novel mechanism: hyperglycemia-induced cardiomyocyte ER stress and cell death are mediated via MCP-1 production and induction of a novel zinc-finger protein MCPIP [109].

**Figure 3:**
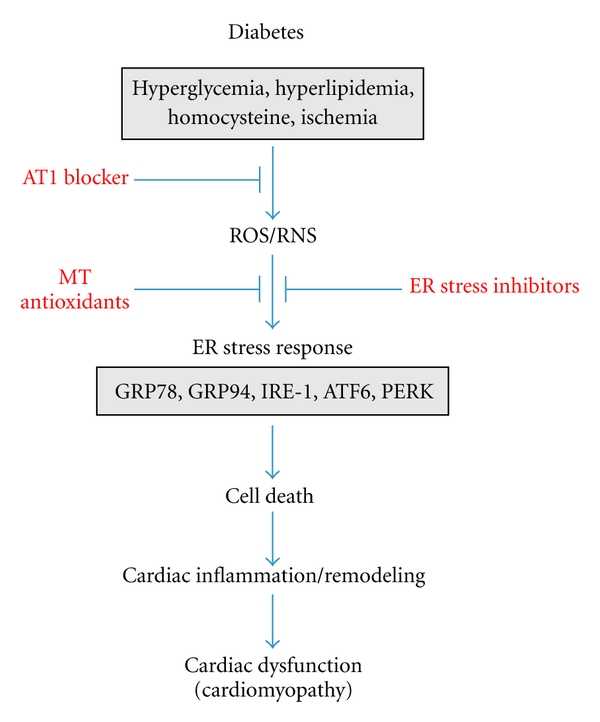
Outlining the involvement of ER stress in the development of diabetic cardiomyopathy.

In summary, various pathogenic factors of diabetes most likely induce ROS that in turns causes the ER stress and associated cell death, as illustrated in Figure 3. Cardiac cell death will initiate the cardiac inflammation and remodeling and eventually cardiac dysfunction, that is, diabetic cardiomyopathy. The pathogenic factors of diabetes include hyperglycemia, hyperlipidemia, and Ang II and so on (Figure 3).

## 6. Potential Prevention of Diabetic Cardiomyopathy by Inhibition of ER Stress

Since the above studies have strongly demonstrated the critical role of ER stress in the development of diabetic cardiomyopathy, ER stress inhibitors would be the new targets for drug discovery and therapeutic intervention in diabetic cardiomyopathy. Chemical or pharmaceutical chaperones, such as 4-phenyl butyric acid (PBA), TUDCA are a group of low-molecular-weight compounds known to stabilize protein conformation to improve ER folding capacity and facilitate the trafficking of mutant proteins. Studies have shown that these chemical ER chaperones can reduce ER stress in a mouse model of type 2 diabetes [112]. TUDCA normalized levels of GRP78 and GRP94 and mitochondrial GSK-3*β* in the rat model of type 2 diabetes [111]. Inhibition of ER stress with tauroursodeoxycholate (TUDC) and PBA resulted in the attenuation of cardiomyoblast death [109]. Our recent studies have shown cardiac overexpression of MT rescued diabetes-, Ang II-, and even chemical ER stressor-induced cardiac cell death via suppression of cardiac ER stress. MT protection against Ang II-induced ER stress and associated apoptotic effects are mediated by its antioxidant action; this suggests that MT inducers such as zinc may also play the same role as the ER stress direct inhibitors [109, 112]. We have treated diabetic mice for the first three months after the establishment of STZ-induced hyperglycemia, resulting in a significant prevention of the development of diabetic cardiomyopathy [113]. Although we did not examine the status of ER stress in the heart for that study, zinc treatment significantly induced cardiac MT expression that might prevent diabetic induction of the ER stress and associated cell death as we observed in other studies [86].

There were also some studies to indirectly inhibit ER stress for the prevention of diabetic cardiomyopathy. Wu et al. have explored the possibility to use valsartan to prevent diabetic ER stress and consequently prevented diabetic cardiomyopathy [99]. They demonstrated that valsartan can ameliorate ER stress-induced cardiac remodeling and myocardial apoptosis in diabetic cardiomyopathy. Similarly, hydrogen sulfide was also explored for the potential to attenuate hyperhomocysteinemia-induced cardiomyocytic ER stress in the rat model [103], with a finding that hydrogen sulfide attenuated cardiomyocyte ER stress in Hcy-induced cardiac injury.

14-3-3 protein was also found to protect against cardiac ER stress and ER stress-initiated apoptosis in experimental diabetes [114]. STZ was used to induce transgenic mice that showed cardiac-specific expression of a dominant negative (DN) 14-3-3eta protein mutant. The expression levels of cardiac GRP78, IRE-1*α*, and TRAF 2 protein were significantly increased in the diabetic DN 14-3-3eta mice compared with the diabetic wild-type control. Moreover, cardiac apoptosis and the expression of CHOP, caspase-12, and cleaved caspase-12 protein were significantly increased in the diabetic DN 14-3-3eta mice. Therefore, they found that partial depletion of 14-3-3 protein in the diabetic heart exacerbates cardiac ER stress and activates ER stress-induced apoptosis pathways, at least in part, through the regulation of CHOP and caspase-12 via the IRE-1*α*/TRAF2 pathway. The enhancement of 14-3-3 protein expression can be used as a novel protective therapy against ER stress and ER stress-initiated apoptosis in the diabetic heart [114].

It is reported that low levels of adiponectin, a fat-derived hormone, were found to be correlated with coronary heart disease, type 2 diabetes, obesity, and insulin resistance. Conversely, high adiponectin levels are predictive of reduced coronary risk in long-term epidemiologic studies. Whether adiponectin has certain cardiac protection and, if so, whether the cardiac protection of adiponectin is related to ER stress have been questioned by Dong and Ren [115]. They have examined the role of adiponectin in cardiac contractile function in the db/db model of diabetic obesity and demonstrated that adiponectin improved cardiomyocyte contractile function in db/db diabetic obese mice, which was not associated with improvement of the ER stress [115]. They demonstrated that cardiomyocytes from db/db mice exhibited greater cross-sectional area, depressed peak shortening, and maximal velocity of shortening/relengthening as well as prolonged duration of relengthening. Consistently, myocytes from db/db mice displayed a reduced electrically stimulated rise in intracellular Ca^2+^ and prolonged intracellular Ca^2+^ decay. These functional and Ca^2+^ changes were abrogated by adiponectin treatment. Levels of the phosphorylated ER stress makers PERK (Thr980), IRE-1, and eIF2*α* were significantly elevated in db/db mice compared with lean controls, but the effect was unaffected by adiponectin. Collectively, they concluded that adiponectin improves cardiomyocyte dysfunction in db/db diabetic obese mice through an ER stress-independent mechanism. However, this study was in contrast to another study that showed that TUDCA normalized levels of GRP78 and GRP94 and mitochondrial GSK-3*β* in the rat model of type 2 diabetes resulted in significant prevention of diabetic cardiac damage [111]. Therefore more studies with different types of diabetes and different conditions are needed to address whether inhibition of ER stress can prevent diabetic cardiomyopathy in T2D model.

As illustrated in Figure 3, these studies have indicated the potential application of various direct and indirect ER stress inhibitors to prevent diabetic ER stress-mediated cell death, and eventually the development of the cardiac remodeling and dysfunction, as a potential approach applied clinically in the future.

## 7. Conclusions

In conclusion, although our understanding of the pathophysiological role of ER stress in diabetic cardiomyopathy has progressed in recent years, many important issues are still unresolved. There are several fundamental questions. One of these important issues is how the cell decides between life and death after the onset of ER stress. Improved understanding of the molecular mechanisms underlying UPR activation and ER-initiated apoptosis in diabetic cardiomyopathy will provide us with new targets for drug discovery and therapeutic intervention.

## References

[1] Sundar Rajan S, Srinivasan V, Balasubramanyam M, Tatu U (2007). Endoplasmic reticulum (ER) stress & diabetes. *Indian Journal of Medical Research*.

[2] Harding HP, Ron D (2002). Endoplasmic reticulum stress and the development of diabetes: a review. *Diabetes*.

[3] Ma Y, Hendershot LM (2001). The unfolding tale of the unfolded protein response. *Cell*.

[4] Wiest DL, Burkhardt JK, Hester S, Hortsch M, Meyer DI, Argon Y (1990). Membrane biogenesis during B cell differentiation: most endoplasmic reticulum proteins are expressed coordinately. *Journal of Cell Biology*.

[5] Iwakoshi NN, Lee AH, Vallabhajosyula P, Otipoby KL, Rajewsky K, Glimcher LH (2003). Plasma cell differentiation and the unfolded protein response intersect at the transcription factor XBP-I. *Nature Immunology*.

[6] Gunn KE, Gifford NM, Mori K, Brewer JW (2004). A role for the unfolded protein response in optimizing antibody secretion. *Molecular Immunology*.

[7] Mori K (2000). Tripartite management of unfolded proteins in the endoplasmic reticulum. *Cell*.

[8] Patil C, Walter P (2001). Intracellular signaling from the endoplasmic reticulum to the nucleus: the unfolded protein response in yeast and mammals. *Current Opinion in Cell Biology*.

[9] Harding HP, Calfon M, Urano F, Novoa I, Ron D (2002). Transcriptional and translational control in the mammalian unfolded protein response. *Annual Review of Cell and Developmental Biology*.

[10] Xu C, Bailly-Maitre B, Reed JC (2005). Endoplasmic reticulum stress: cell life and death decisions. *Journal of Clinical Investigation*.

[11] Zhang K, Kaufman RJ (2006). The unfolded protein response: a stress signaling pathway critical for health and disease. *Neurology*.

[12] Lee AS, Delegeane AM, Baker V, Chow PC (1983). Transcriptional regulation of two genes specifically induced by glucose starvation in a hamster mutant fibroblast cell line. *Journal of Biological Chemistry*.

[13] Ma Y, Hendershot LM (2002). The mammalian endoplasmic reticulum as a sensor for cellular stress. *Cell Stress and Chaperones*.

[14] Rutkowski DT, Kaufman RJ (2004). A trip to the ER: coping with stress. *Trends in Cell Biology*.

[15] Sidrauski C, Chapman R, Walter P (1998). The unfolded protein response: an intracellular signalling pathway with many surprising features. *Trends in Cell Biology*.

[16] Hampton RY (2000). ER stress response: getting the UPR hand on misfolded proteins. *Current Biology*.

[17] Shen X, Zhang K, Kaufman RJ (2004). The unfolded protein response: a stress signaling pathway of the endoplasmic reticulum. *Journal of Chemical Neuroanatomy*.

[18] Avogaro A, Vigili De Kreutzenberg S, Negut C, Tiengo A, Scognamiglio R (2004). Diabetic cardiomyopathy: a metabolic perspective. *American Journal of Cardiology*.

[19] Picano E (2003). Diabetic cardiomyopathy: the importance of being earliest. *Journal of the American College of Cardiology*.

[20] Dyntar D, Sergeev P, Klisic J, Ambühl P, Schaub MC, Donath MY (2006). High glucose alters cardiomyocyte contacts and inhibits myofibrillar formation. *Journal of Clinical Endocrinology and Metabolism*.

[21] Feuvray D (2004). Diabetic cardiomyopathy. *Archives des Maladies du Coeur et des Vaisseaux*.

[22] Tappia PS, Asemu G, Aroutiounova N, Dhalla NS (2004). Defective sarcolemmal phospholipase C signaling in diabetic cardiomyopathy. *Molecular and Cellular Biochemistry*.

[23] Ligeti L, Szenczi O, Prestia CM (2006). Altered calcium handling is an early sign of streptozotocin-induced diabetic cardiomyopathy. *International Journal of Molecular Medicine*.

[24] Pereira L, Matthes J, Schuster I (2006). Mechanisms of [Ca^2+^]_i_ transient decrease in cardiomyopathy of *db/db* type 2 diabetic mice. *Diabetes*.

[25] Galicka-Latała D, Konduracka E, Kuźniewski M, Fedak D, Sieradzki J (2005). Myocardial dysfunction, neuropathy and nephropathy in long standing type 1 diabetic patients. *Przegląd Lekarski*.

[26] An D, Rodrigues B (2006). Role of changes in cardiac metabolism in development of diabetic cardiomyopathy. *American Journal of Physiology*.

[27] Adeghate E (2004). Molecular and cellular basis of the aetiology and management of diabetic cardiomyopathy: a short review. *Molecular and Cellular Biochemistry*.

[28] Cai L, Li W, Wang G, Guo L, Jiang Y, James Kang Y (2002). Hyperglycemia-induced apoptosis in mouse myocardium: mitochondrial cytochrome c-mediated caspase-3 activation pathway. *Diabetes*.

[29] Cai L, Kang YJ (2003). Cell death and diabetic cardiomyopathy. *Cardiovascular Toxicology*.

[30] Cai L, Wang Y, Zhou G (2006). Attenuation by metallothionein of early cardiac cell death via suppression of mitochondrial oxidative stress results in a prevention of diabetic cardiomyopathy. *Journal of the American College of Cardiology*.

[31] Ghosh S, Rodrigues B (2006). Cardiac cell death in early diabetes and its modulation by dietary fatty acids. *Biochimica et Biophysica Acta*.

[32] Li Z, Zhang T, Dai H (2007). Involvement of endoplasmic reticulum stress in myocardial apoptosis of streptozocin-induced diabetic rats. *Journal of Clinical Biochemistry and Nutrition*.

[33] Li Z, Zhang T, Dai H (2008). Endoplasmic reticulum stress is involved in myocardial apoptosis of streptozocin-induced diabetic rats. *Journal of Endocrinology*.

[34] Tirasophon W, Welihinda AA, Kaufman RJ (1998). A stress response pathway from the endoplasmic reticulum to the nucleus requires a novel bifunctional protein kinase/endoribonuclease (Ire1p) in mammalian cells. *Genes and Development*.

[35] Wang XZ, Harding HP, Zhang Y, Jolicoeur EM, Kuroda M, Ron D (1998). Cloning of mammalian Ire1 reveals diversity in the ER stress responses. *The EMBO Journal*.

[36] Haze K, Yoshida H, Yanagi H, Yura T, Mori K (1999). Mammalian transcription factor ATF6 is synthesized as a transmembrane protein and activated by proteolysis in response to endoplasmic reticulum stress. *Molecular Biology of the Cell*.

[37] Shi Y, Vattem KM, Sood R (1998). Identification and characterization of pancreatic eukaryotic initiation factor 2 *α*-subunit kinase, PEK, involved in translational control. *Molecular and Cellular Biology*.

[38] Harding HP, Zhang Y, Ron D (1999). Protein translation and folding are coupled by an endoplasmic-reticulum-resident kinase. *Nature*.

[39] Bertolotti A, Zhang Y, Hendershot LM, Harding HP, Ron D (2000). Dynamic interaction of BiP and ER stress transducers in the unfolded-protein response. *Nature Cell Biology*.

[40] Ma K, Vattem KM, Wek RC (2002). Dimerization and release of molecular chaperone inhibition facilitate activation of eukaryotic initiation factor-2 kinase in response to endoplasmic reticulum stress. *Journal of Biological Chemistry*.

[41] Ma Y, Hendershot LM (2003). Delineation of a negative feedback regulatory loop that controls protein translation during endoplasmic reticulum stress. *Journal of Biological Chemistry*.

[42] Thoma C, Bergamini G, Galy B, Hundsdoerfer P, Hentze MW (2004). Enhancement of IRES-mediated translation of the c-myc and BiP mRNAs by the poly(A) tail is independent of intact eIF4G and PABP. *Molecular Cell*.

[43] Lu PD, Harding HP, Ron D (2004). Translation reinitiation at alternative open reading frames regulates gene expression in an integrated stress response. *Journal of Cell Biology*.

[44] Shamu CE, Walter P (1996). Oligomerization phosphorylation of the Ire1p kinase during intracellular signaling from the endoplasmic reticulum to the nucleus. *The EMBO Journal*.

[45] Sidrauski C, Walter P (1997). The transmembrane kinase Ire1p is a site-specific endonuclease that initiates mRNA splicing in the unfolded protein response. *Cell*.

[46] Lee AH, Iwakoshi NN, Glimcher LH (2003). XBP-1 regulates a subset of endoplasmic reticulum resident chaperone genes in the unfolded protein response. *Molecular and Cellular Biology*.

[47] Zhu C, Johansen FE, Prywes R (1997). Interaction of ATF6 and serum response factor. *Molecular and Cellular Biology*.

[48] Yoshida H, Haze K, Yanagi H, Yura T, Mori K (1998). Identification of the cis-acting endoplasmic reticulum stress response element responsible for transcriptional induction of mammalian glucose-regulated proteins: involvement of basic leucine zipper transcription factors. *Journal of Biological Chemistry*.

[49] Shen J, Snapp EL, Lippincott-Schwartz J, Prywes R (2005). Stable binding of ATF6 to BiP in the endoplasmic reticulum stress response. *Molecular and Cellular Biology*.

[50] Shen J, Chen X, Hendershot L, Prywes R (2002). ER stress regulation of ATF6 localization by dissociation of BiP/GRP78 binding and unmasking of golgi localization signals. *Developmental Cell*.

[51] Thuerauf DJ, Arnold ND, Zechner D (1998). p38 mitogen-activated protein kinase mediates the transcriptional induction of the atrial natriuretic factor gene through a serum response element: a potential role for the transcription factor ATF6. *Journal of Biological Chemistry*.

[52] Wang Y, Shen J, Arenzana N, Tirasophon W, Kaufman RJ, Prywes R (2000). Activation of ATF6 and an ATF6 DNA binding site by the endoplasmic reticulum stress response. *Journal of Biological Chemistry*.

[53] Yoshida H, Okada T, Haze K (2000). ATF6 activated by proteolysis binds in the presence of NF-Y (CBF) directly to the cis-acting element responsible for the mammalian unfolded protein response. *Molecular and Cellular Biology*.

[54] Li MQ, Baumeister P, Roy B (2000). ATF6 as a transcription activator of the endoplasmic reticulum stress element: thapsigargin stress-induced changes and synergistic interactions with NF-Y and YY1. *Molecular and Cellular Biology*.

[55] Yoshida H, Okada T, Haze K (2001). Endoplasmic reticulum stress-induced formation of transcription factor complex ERSF including NF-Y (CBF) and activating transcription factors 6*α* and 6*β* that activates the mammalian unfolded protein response. *Molecular and Cellular Biology*.

[56] Kokame K, Kato H, Miyata T (2001). Identification of ERSE-II, a new cis-acting element responsible for the ATF6-dependent mammalian unfolded protein response. *Journal of Biological Chemistry*.

[57] Parker R, Phan T, Baumeister P (2001). Identification of TFII-I as the endoplasmic reticulum stress response element binding factor ERSF: its autoregulation by stress and interaction with ATF6. *Molecular and Cellular Biology*.

[58] Ma Y, Hendershot LM (2004). Herp is dually regulated by both the endoplasmic reticulum stress-specific branch of the unfolded protein response and a branch that is shared with other cellular stress pathways. *Journal of Biological Chemistry*.

[59] Glembotski CC (2007). Endoplasmic reticulum stress in the heart. *Circulation Research*.

[60] Yoneda T, Imaizumi K, Oono K (2001). Activation of caspase-12, an endoplastic reticulum (ER) resident caspase, through tumor necrosis factor receptor-associated factor 2-dependent mechanism in response to the ER stress. *Journal of Biological Chemistry*.

[61] Nishitoh H, Matsuzawa A, Tobiume K (2002). ASK1 is essential for endoplasmic reticulum stress-induced neuronal cell death triggered by expanded polyglutamine repeats. *Genes and Development*.

[62] Nakagawa T, Zhu H, Morishima N (2000). Caspase-12 mediates endoplasmic-reticulum-specific apoptosis and cytotoxicity by amyloid-*β*. *Nature*.

[63] Nakagawa T, Yuan J (2000). Cross-talk between two cysteine protease families: activation of caspase-12 by calpain in apoptosis. *Journal of Cell Biology*.

[64] Rao RV, Hermel E, Castro-Obregon S (2001). Coupling endoplasmic reticulum stress to the cell death program. Mechanism of caspase activation. *Journal of Biological Chemistry*.

[65] Morishima N, Nakanishi K, Takenouchi H, Shibata T, Yasuhiko Y (2002). An endoplasmic reticulum stress-specific caspase cascade in apoptosis. Cytochrome c-independent activation of caspase-9 by caspase-12. *Journal of Biological Chemistry*.

[66] Boyce M, Yuan J (2006). Cellular response to endoplasmic reticulum stress: a matter of life or death. *Cell Death and Differentiation*.

[67] Ma Y, Brewer JW, Alan Diehl J, Hendershot LM (2002). Two distinct stress signaling pathways converge upon the CHOP promoter during the mammalian unfolded protein response. *Journal of Molecular Biology*.

[68] Wang XZ, Lawson B, Brewer JW (1996). Signals from the stressed endoplasmic reticulum induce C/EBP-homologous protein (CHOP/GADD153). *Molecular and Cellular Biology*.

[69] Harding HP, Zhang Y, Zeng H (2003). An integrated stress response regulates amino acid metabolism and resistance to oxidative stress. *Molecular Cell*.

[70] Pahl HL, Baeuerle PA (1997). The ER overload response: activation of NF-*κ*B. *Trends in Biochemical Sciences*.

[71] Buytaert E, Callewaert G, Hendrickx N (2006). Role of endoplasmic reticulum depletion and multidomain proapoptotic BAX and BAK proteins in shaping cell death after hypericin-mediated photodynamic therapy. *FASEB Journal*.

[72] Mao C, Tai WC, Bai Y, Poizat C, Lee AS (2006). In vivo regulation of Grp78/BiP transcription in the embryonic heart: role of the endoplasmic reticulum stress response element and GATA-4. *Journal of Biological Chemistry*.

[73] Luo S, Mao C, Lee B, Lee AS (2006). GRP78/BiP is required for cell proliferation and protecting the inner cell mass from apoptosis during early mouse embryonic development. *Molecular and Cellular Biology*.

[74] Wanderling S, Simen BB, Ostrovsky O (2007). GRP94 is essential for mesoderm induction and muscle development because it regulates insulin-like growth factor secretion. *Molecular Biology of the Cell*.

[75] Masaki T, Yoshida M, Noguchi S (1999). Targeted disruption of CRE-Binding factor TREB5 gene leads to cellular necrosis in cardiac myocytes at the embryonic stage. *Biochemical and Biophysical Research Communications*.

[76] Pan Y-X, Lin L, Ren A-J (2004). HSP70 and GRP78 induced by endothelin-1 pretreatment enhance tolerance to hypoxia in cultured neonatal rat cardiomyocytes. *Journal of Cardiovascular Pharmacology*.

[77] Fu HY, Minamino T, Tsukamoto O (2008). Overexpression of endoplasmic reticulum-resident chaperone attenuates cardiomyocyte death induced by proteasome inhibition. *Cardiovascular Research*.

[78] Vitadello M, Penzo D, Petronilli V (2003). Overexpression of the stress protein Grp94 reduces cardiomyocyte necrosis due to calcium overload and simulated ischemia. *The FASEB Journal*.

[79] Martindale JJ, Fernandez R, Thuerauf D (2006). Endoplasmic reticulum stress gene induction and protection from ischemia/reperfusion injury in the hearts of transgenic mice with a tamoxifen-regulated form of ATF6. *Circulation Research*.

[80] Petrovski G, Das S, Juhasz B, Kertesz A, Tosaki A, Das DK (2011). Cardioprotection by endoplasmic reticulum stress-induced autophagy. *Antioxidants and Redox Signaling*.

[81] Azfer A, Niu J, Rogers LM, Adamski FM, Kolattukudy PE (2006). Activation of endoplasmic reticulum stress response during the development of ischemic heart disease. *American Journal of Physiology*.

[82] Hamada H, Suzuki M, Yuasa S (2004). Dilated cardiomyopathy caused by aberrant endoplasmic reticulum quality control in mutant KDEL receptor transgenic mice. *Molecular and Cellular Biology*.

[83] Shohet RV, Garcia JA (2007). Keeping the engine primed: HIF factors as key regulators of cardiac metabolism and angiogenesis during ischemia. *Journal of Molecular Medicine*.

[84] Groenendyk J, Sreenivasaiah PK, Kim DH, Agellon LB, Michalak M (2010). Biology of endoplasmic reticulum stress in the heart. *Circulation Research*.

[85] Scarabelli TM, Gottlieb RA (2004). Functional and clinical repercussions of myocyte apoptosis in the multifaceted damage by ischemia/reperfusion injury: old and new concepts after 10 years of contributions. *Cell Death and Differentiation*.

[86] Xu J, Wang G, Wang Y (2009). Diabetes- and angiotensin II-induced cardiac endoplasmic reticulum stress and cell death: metallothionein protection. *Journal of Cellular and Molecular Medicine*.

[87] Nakatani Y, Kaneto H, Kawamori D (2005). Involvement of endoplasmic reticulum stress in insulin resistance and diabetes. *Journal of Biological Chemistry*.

[88] Hayden MR, Tyagi SC, Kerklo MM, Nicolls MR (2005). Type 2 diabetes mellitus as a conformational disease. *Journal of the Pancreas*.

[89] Okada KI, Minamino T, Tsukamoto Y (2004). Prolonged endoplasmic reticulum stress in hypertrophic and failing heart after aortic constriction: possible contribution of endoplasmic reticulum stress to cardiac myocyte apoptosis. *Circulation*.

[90] Minamino T, Kitakaze M (2010). ER stress in cardiovascular disease. *Journal of Molecular and Cellular Cardiology*.

[91] Mao W, Fukuoka S, Iwai C (2007). Cardiomyocyte apoptosis in autoimmune cardiomyopathy: mediated via endoplasmic reticulum stress and exaggerated by norepinephrine. *American Journal of Physiology*.

[92] Li SY, Gilbert SAB, Li Q, Ren J (2009). Aldehyde dehydrogenase-2 (ALDH2) ameliorates chronic alcohol ingestion-induced myocardial insulin resistance and endoplasmic reticulum stress. *Journal of Molecular and Cellular Cardiology*.

[93] Nickson P, Toth A, Erhardt P (2007). PUMA is critical for neonatal cardiomyocyte apoptosis induced by endoplasmic reticulum stress. *Cardiovascular Research*.

[94] Toth A, Jeffers JR, Nickson P (2006). Targeted deletion of Puma attenuates cardiomyocyte death and improves cardiac function during ischemia-reperfusion. *American Journal of Physiology*.

[95] Jackson CV, McGrath GM, Tahiliani AG (1985). A functional and ultrastructural analysis of experimental diabetic rat myocardium. Manifestation of a cardiomyopathy. *Diabetes*.

[96] Bhimji S, Godin DV, McNeill JH (1986). Myocardial ultrastructural changes in alloxan-induced diabetes in rabbits. *Acta Anatomica*.

[97] Zhou G, Li X, Hein DW (2008). Metallothionein suppresses angiotensin II-induced nicotinamide adenine dinucleotide phosphate oxidase activation, nitrosative stress, apoptosis, and pathological remodeling in the diabetic heart. *Journal of the American College of Cardiology*.

[98] Sukumaran V, Watanabe K, Veeraveedu PT (2011). Olmesartan, an AT1 antagonist, attenuates oxidative stress, endoplasmic reticulum stress and cardiac inflammatory mediators in rats with heart failure induced by experimental autoimmune myocarditis. *International Journal of Biological Sciences*.

[99] Wu T, Dong Z, Geng J (2011). Valsartan protects against ER stress-induced myocardial apoptosis via CHOP/Puma signaling pathway in streptozotocin-induced diabetic rats. *European Journal of Pharmaceutical Sciences*.

[100] Beer S, Golay S, Bardy D (2005). Increased plasma levels of N-terminal brain natriuretic peptide (NT-proBNP) in type 2 diabetic patients with vascular complications. *Diabetes and Metabolism*.

[101] Siebenhofer A, Ng LL, Plank J, Berghold A, Hödl R, Pieber TR (2003). Plasma N-terminal pro-brain natriuretic peptide in type 1 diabetic patients with and without diabetic nephropathy. *Diabetic Medicine*.

[102] Ganguly PK (1991). Role of atrial natriuretic peptide in congestive heart failure due to chronic diabetes. *Canadian Journal of Cardiology*.

[103] Wei H, Zhang R, Jin H (2010). Hydrogen sulfide attenuates hyperhomocysteinemia-induced cardiomyocytic endoplasmic reticulum stress in rats. *Antioxidants and Redox Signaling*.

[104] Cai L (2006). Suppression of nitrative damage by metallothionein in diabetic heart contributes to the prevention of cardiomyopathy. *Free Radical Biology and Medicine*.

[105] Cai L, Satoh M, Tohyama C, Cherian MG (1999). Metallothionein in radiation exposure: its induction and protective role. *Toxicology*.

[106] Cai L, Klein JB, Kang YJ (2000). Metallothionein inhibits peroxynitrite-induced DNA and lipoprotein damage. *Journal of Biological Chemistry*.

[107] Cai L, Wang J, Li Y (2005). Inhibition of superoxide generation and associated nitrosative damage is involved in metallothionein prevention of diabetic cardiomyopathy. *Diabetes*.

[108] Wang Y, Feng W, Xue W (2009). Inactivation of GSK-3*β* by metallothionein prevents diabetes-related changes in cardiac energy metabolism, inflammation, nitrosative damage, and remodeling. *Diabetes*.

[109] Younce CW, Wang K, Kolattukudy PE (2010). Hyperglycaemia-induced cardiomyocyte death is mediated via MCP-1 production and induction of a novel zinc-finger protein MCPIP. *Cardiovascular Research*.

[110] Li J, Zhu H, Shen E, Wan L, Arnold JMO, Peng T (2010). Deficiency of Rac1 blocks NADPH oxidase activation, inhibits endoplasmic reticulum stress, and reduces myocardial remodeling in a mouse model of type 1 diabetes. *Diabetes*.

[111] Miki T, Miura T, Hotta H (2009). Endoplasmic reticulum stress in diabetic hearts abolishes erythropoietin-induced myocardial protection by impairment of phospho-glycogen synthase kinase-3*β*-mediated suppression of mitochondrial permeability transition. *Diabetes*.

[112] Özcan U, Yilmaz E, Özcan L (2006). Chemical chaperones reduce ER stress and restore glucose homeostasis in a mouse model of type 2 diabetes. *Science*.

[113] Wang J, Song Y, Elsherif L (2006). Cardiac metallothionein induction plays the major role in the prevention of diabetic cardiomyopathy by zinc supplementation. *Circulation*.

[114] Sari FR, Watanabe K, Thandavarayan RA (2010). 14-3-3 protein protects against cardiac endoplasmic reticulum stress (ERS) and ERS-initiated apoptosis in experimental diabetes. *Journal of Pharmacological Sciences*.

[115] Dong F, Ren J (2009). Adiponectin improves cardiomyocyte contractile fnction in db/db diabetic obese mice. *Obesity*.

